# Compassionate Health Care

**DOI:** 10.3390/healthcare11010109

**Published:** 2022-12-30

**Authors:** Maria Malliarou

**Affiliations:** Laboratory of Education and Research of Trauma Care and Patient Safety (Labedu TraumaCare), Faculty of Nursing, University of Thessaly, Gaiopolis, Larissa-Trikala Ring Road, 41100 Larissa, Greece; malliarou@uth.gr

Compassionate care is a key component in nursing and midwifery quality care [1]. Compassion is a very complex term, which primarily includes other equally important values, such as sympathy, empathy, and respect. It concerns a deep awareness of the situation that a person is suffering, combined with the will to provide relief [2]. An International Online Survey Involving 15 Countries demonstrated a shared understanding of the importance of compassion as well as some common perceptions of the attributes of compassionate care [3].

Compassion also plays an important role in facilitating patient-centered communication, in cases where the nurse is important to be aware of what has to do with the patient’s philosophy and preferences, when to report bad news to patients, or when to offer palliative care [4].

Some researchers believe that treating oneself and others with compassion promotes individual well-being and improves mental health [5]. If this is applied in the hospital environment, then it has practical advantages. Modern science holds compassion as a necessary skill in order for the health professional to provide proper care to patients. Identification of the emotional state of others and a relatively understanding response, together with the desire to relieve the pain others experience, are important aspects of quality healthcare. However, the caregiver must be able to manage his own emotions so that his own health status is not endangered by psychiatric or psychological disorders, such as compassion fatigue. Healthcare leaders especially need to adopt compassionate methods of leadership [6] to educate their personnel in communication skills and social issues, and to value, support, and reward compassionate methods of communication with patients, family, and caregivers. Compassionate care, which has been proven as a necessity in healthcare, is hampered by certain factors, some of which can be attributed to the work environment itself [7]. An organization’s culture refers to the behaviors of its members and these in turn express the attitudes, beliefs, and values that have been defined by the leadership team as an integral component of its operation. Promoting a compassionate culture can render benefits such as good fellowship, professional satisfaction, and improved health services [8].

In conclusion, compassion can create an environment of safety for patients and nurse professionals. It is fundamental in nursing care and can build trust and good relationships between all parties involved in the care of the patient.
